# Genome sequences of five *Klebsiella* bacteriophages that belong to the genus *Jiaodavirus*

**DOI:** 10.1128/mra.01056-24

**Published:** 2024-11-22

**Authors:** Tracey L. Peters, Caitlin D. Urick, Martin Georges, Kevin A. Burke, Olga A. Kirillina, Nino Mzhavia, Lillian Musila, Andrey A. Filippov, Mikeljon P. Nikolich

**Affiliations:** 1 Institute for Modeling Collaboration and Innovation, University of Idaho, Moscow, Idaho, USA; 2 Wound Infections Department, Bacterial Diseases Branch, Walter Reed Army Institute of Research (WRAIR), Silver Spring, Maryland, USA; 3 Department of Emerging Infectious Diseases, Walter Reed Army Institute of Research-Africa (WRAIR-Africa), Nairobi, Kenya; Queens College Department of Biology, Queens, New York, USA

**Keywords:** *Klebsiella pneumoniae*, bacteriophages, *Straboviridae*, *Jiaodavirus*, whole genomes, therapeutic candidates

## Abstract

We describe the genomes of five lytic *Klebsiella pneumoniae* myophages, therapeutic candidates, that belong to the family *Straboviridae* and genus *Jiaodavirus*. The genomes ranged from 165,574 to 169,768 bp, with ca. 40% GC content, contained 289–300 coding sequences, had 15–16 tRNA genes, and no terminal repeats.

## ANNOUNCEMENT

Growing multidrug resistance of *Klebsiella pneumoniae* infections promotes studies on phages as alternative antibacterials. Phages have shown efficacy against drug-resistant *K. pneumoniae* infections in humans (1–3). Our team is harvesting *K. pneumoniae* phages in the USA and Kenya for therapeutic cocktails. Here we report the genomes of phages vB_Kpn11382-KEN22 (KEN22), vB_Kpn529046-KEN25-1 (KEN25-1), vB_Kpn529046-KEN25-2 (KEN25-2), vB_Kpn529046-KEN37 (KEN37), and vB_Kpn529046-KEN39 (KEN39).

The phages were isolated from sewage, fresh water, or Indian Ocean water collected from March to April 2021 in Nairobi and Mombasa, Kenya, for enrichment using human isolates of *K. pneumoniae* MRSN 11382 (ST17) and MRSN 529046 (ST34) (Table 1). After the enrichment procedure (4), phages were isolated from single plaques three times on double-layered heart infusion broth (HIB) agar (1.5%/0.7%), propagated in HIB with shaking (37°C/120 rpm), and their DNA was extracted using the QIAamp DNA Mini Kit (Qiagen, Germantown, MD) (4). Libraries were prepared with the KAPA HyperPlus Kit (Roche Diagnostics, Indianapolis, IN) and sequenced on an Illumina MiSeq (Illumina, Inc., San Diego, CA) with the MiSeq Reagent Kit v3 (600 cycles, 300 bp reads). The quality of paired-end reads (see Table 1) was assessed, and they were trimmed using Fastp (5) v0.22.0. The genomes were assembled with Unicycler (6) v0.5.0; the termini were mapped using PhageTerm (7) v1.0.12; phage lifestyle was predicted with BACPHLIP (8) v0.9.3; protein coding sequences (CDSs) were annotated using the Pharokka pipeline (9–19) v1.4.0; and protein sequences were aligned using DIAMOND (20, 21) v2.0.4. Default parameters were used in all software.

**TABLE 1:** Characteristics of *K. pneumoniae* phages described in this work

Phage ID	Location	Coordinates	Sample source	Enrichment strain	Genome length, bp	G+C (%)	No. of CDSs	No. of tRNA genes	Genome coverage (×)	No. of raw reads	GenBank Accession no.	SRA accession no.
KEN22	Nairobi	−1.366376, 36.7289007	Raw sewage	MRSN 11382	166,645	39.55	294	16	283.6	173,080	PP723050	SRR28813169
KEN25-1	Nairobi	−1.366376, 36.7289007	Raw sewage	MRSN 529046	169,768	39.58	300	15	304.6	983,866	PP723049	SRR28813168
KEN25-2	Nairobi	−1.366376, 36.7289007	Raw sewage	MRSN 529046	165,574	39.59	289	16	561.6	1,080,110	PP723048	SRR28813167
KEN37	Nairobi	−1.263928, 36.879514	Fresh water	MRSN 529046	166,503	39.64	293	16	609.8	650,186	PP723047	SRR28813166
KEN39	Mombasa	−4.037969, 39.727265	Ocean water	MRSN 529046	166,254	39.59	295	15	381.1	252,352	PP723046	SRR28813165

**Fig 1:**
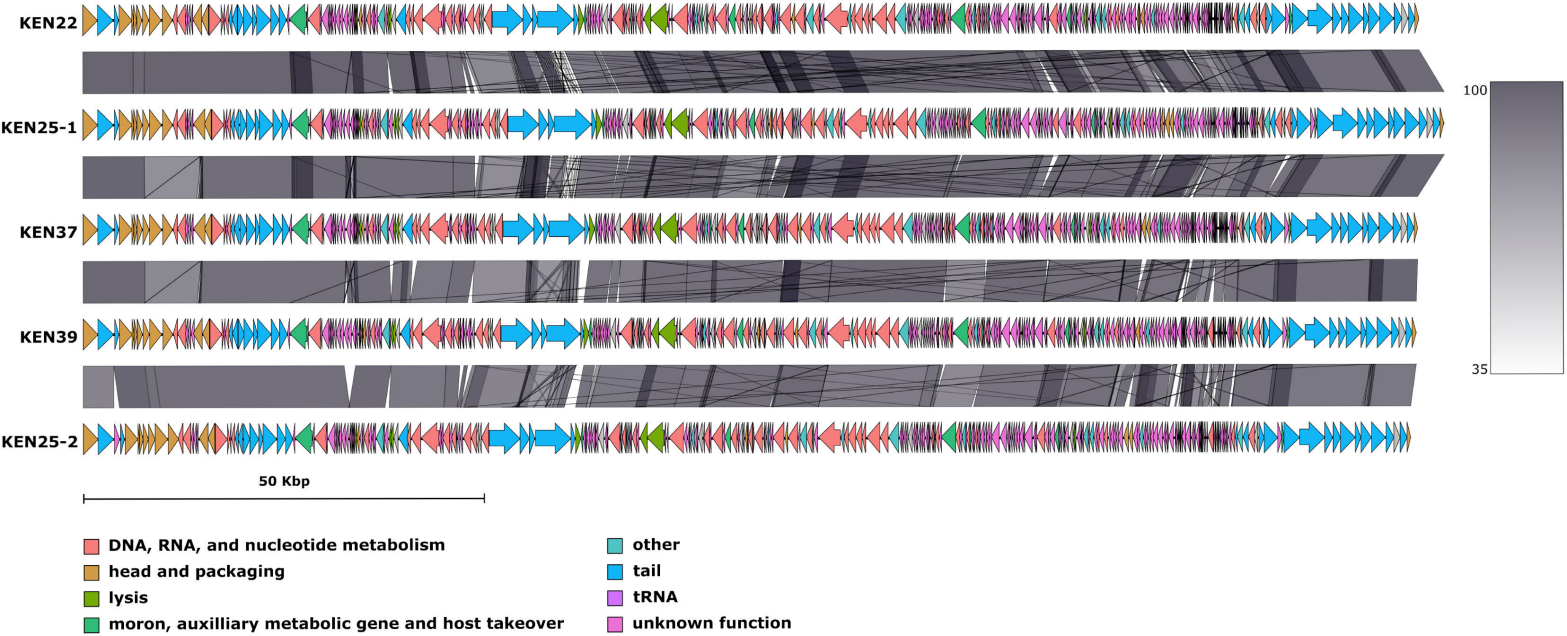
Genome organization of five *Jiaodavirus* phages isolated in Kenya. The genome maps were created using GenoFig v1.1 (https://forgemia.inra.fr/public-pgba/genofig).

The read coverages ranged between 284× and 610×. The genomes of phages KEN22, KEN25-1, KEN25-2, KEN37, and KEN39 varied from 165,574 to 169,768 bp, with 39.55–39.64% GC content, contained between 289 and 300 predicted CDSs, 15–16 tRNA genes (see Table 1; Fig. 1), and no direct terminal repeats were found. Using Mash alignment (19) against the INPHARED database (19), these phages were classified into the family *Straboviridae*, subfamily *Tevenvirinae*, and genus *Jiaodavirus* and demonstrated 88–92% whole-genome average nucleotide identity (cutoff ≥80.75%) (22) with reference genomes of all six species included in this genus (https://ictv.global/taxonomy, retrieved 17 October 2024) and 88–95% identity with 49 other *Jiaodavirus* phages in the NCBI database. Phages of this group have been isolated on *K. pneumoniae*, *Klebsiella oxytoca*, and *Salmonella enterica*. They have myovirus morphology, are lytic, and have relatively broad host ranges. Their genomes are terminally redundant, circularly permuted, and headful-packaged, but these phages degrade host DNA and use the degradation products to synthesize their own genomes, which makes transduction of bacterial genes unlikely (23–28).

BACPHLIP scored KEN22, KEN25-1, KEN25-2, KEN37, and KEN39 genomes at 96% for a virulent life cycle (8). No homology was found between putative proteins of these phages and products associated with lysogenicity, gene transfer, and bacterial proteins including antibiotic resistance determinants (14) and virulence factors (15). Thus, these five phages appear to be lytic and promising as therapeutics.

## Data Availability

The KEN22, KEN25-1, KEN25-2, KEN37, and KEN39 genome BioProject accession number is PRJNA1103181. BioSample accession numbers are SAMN41046533, SAMN41046534, SAMN41046535, SAMN41046536, and SAMN41046537, respectively. GenBank accession numbers and the NCBI Sequence Read Archive accession numbers are listed in Table 1.
